# Patent Foramen Ovale in Children: A Review of Recent Progress

**DOI:** 10.1007/s00246-024-03526-5

**Published:** 2024-06-01

**Authors:** Tingting Zhang, Chao Gao, Wei Chen, Hui Ma, Ling Tao

**Affiliations:** 1 Department of Cardiology, Xijing Hospital, Fourth Military Medical University, Changle West Road, Xi’an, 710032 China; 2 Department of Ultrasound Diagnostics, Xijing Hospital, Fourth Military Medical University, Xi’an, China

**Keywords:** Patent foramen ovale, Children, Pediatric, Stroke, Migraine

## Abstract

The support has been provided by clinical trials and guidelines for managing patent foramen ovale (PFO) in adults; however, the optimal approach is still unclear for treating PFO in pediatric patients. PFO and its associated clinical syndromes, imaging diagnosis, and management in pediatric patients were analyzed by a comprehensive analysis. Extensive research was performed using electronic databases, including PubMed, Cochrane, Web of Science, and EMBASE. This review includes the studies published until February 1st, 2024. A total of 583 articles were obtained, of which 54 were included in the comprehensive review. Numerous evidences have indicated that a right-to-left shunt through a PFO may be involved in cryptogenic stroke in children, although the connection between migraine and aura has not been substantiated by robust evidence. Children with sickle cell disease and a PFO were at higher risks of paradoxical embolization, rare syndromes caused by PFO could also occur in children such as platypnea–orthodeoxia syndrome, myocardial infarction, and decompression sickness. Contrast transthoracic echocardiography was deemed the most appropriate examination for children due to its favorable transthoracic windows, eliminating the need for anesthesia. This review suggested that the additional treatment was not needed as no evidence was provided for potential future complications linked to isolated PFO in children. For children facing unique circumstances related to PFO, a customized interdisciplinary consultation is essential prior to considering medical interventions.

## Introduction

**Fig. 1:**
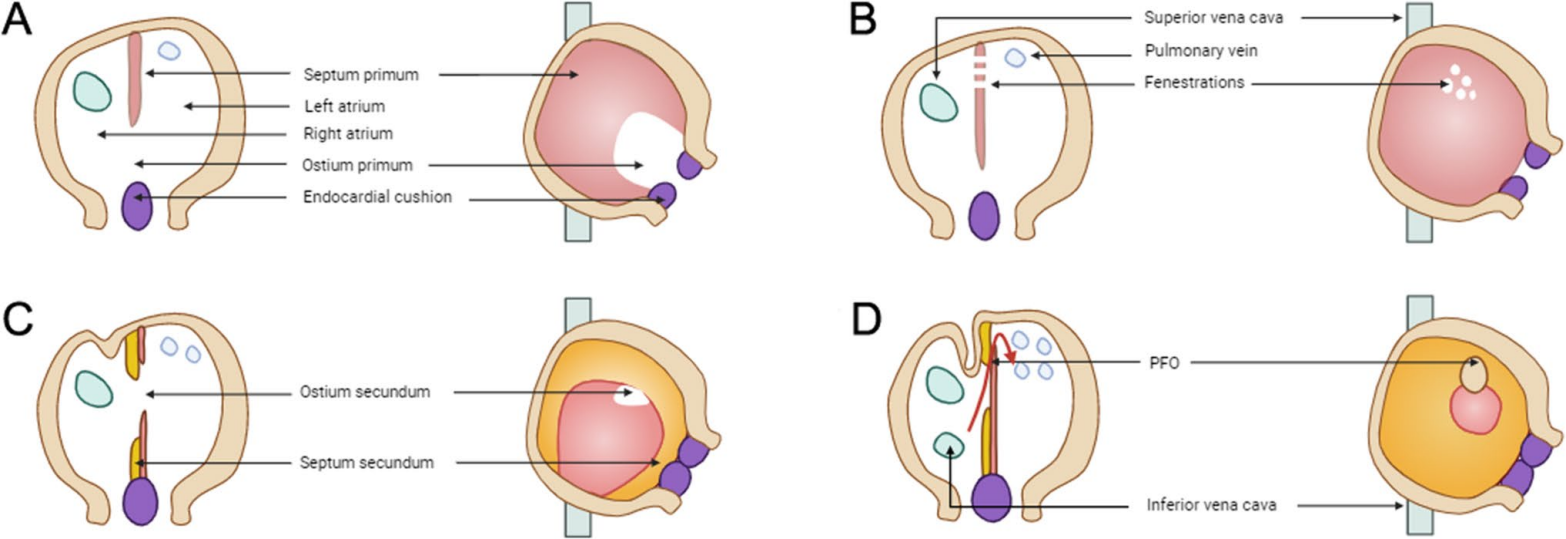
Development of the atrial septal during the embryonic period. **A** The septum primum originates from the posterior superior wall of the common atrium and extends toward the atrioventricular (AV) valves. **B** Fenestrations form in the septum primum and merge together superiorly and anteriorly, while maintaining a connection as fusion occurs inferiorly with the endocardial cushions. **C** Subsequently, the septum secundum emerges on the right atrial side of the septum primum and grows toward the AV valves. The ostium secundum serves as a pathway for the right-to-left shunt of oxygenated blood. **D** The primum and secundum septa do not fuse completely, resulting in a patent foramen ovale (PFO) located at the anterosuperior edge of the fossa ovalis

**Fig. 2:**
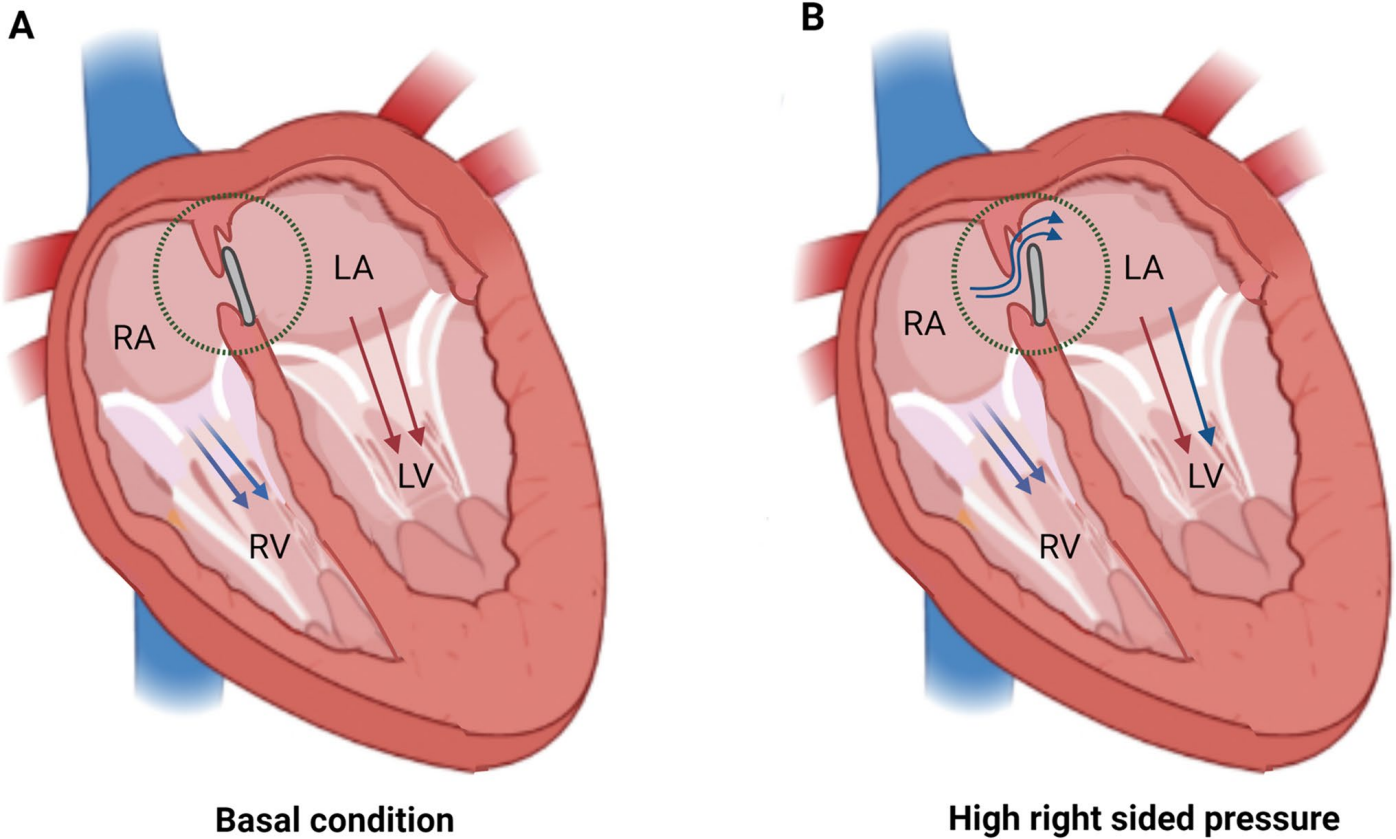
The basal condition **A** and the high right atrial pressure condition **B** of patent foramen ovale (PFO)

A patent foramen ovale (PFO) is a natural connection between the right and left atrial chambers that occurs during fetal development. The embryology of PFO is depicted in Fig. 1 [1]. In the majority of individuals, this connection gradually closes after birth. However, if the primary septum fails to fully fuse with the secondary septum, the PFO remains open in approximately 25% of adults. Under certain conditions that raise the pressure in the right atrium, the right-to-left shunt (RLS) through the PFO (as shown in Fig. 2) can be amplified, thereby increasing the risk of paradoxical embolization (PE).

Clinical trials and guidelines have offered assistance in managing PFO in cryptogenic stroke (CS) among adults [2–4], while the involvement of PFO in migraines among adults remains a subject of debate [5, 6]. Nevertheless, as these trials did not include children, a conclusive determination regarding the role of PFO in pediatric cases is still pending.

Hence, the objective of this current research was to perform an in-depth analysis to assess the relationship between a PFO and different clinical syndromes in children such as CS, migraine, transient ischemic attack (TIA), and other rare clinical syndromes. Furthermore, we investigated the significance of imaging in the detection of PFO in children and delved into the strategies for managing PFO in children.

## Methods

### Data Source and Search Strategy

An electronic PubMed, Cochrane, Web of Science, and EMBASE search were performed by two researchers (TTZ, CG) using the search terms (“patent foramen ovale”) AND (“children” OR “pediatric” OR “adolescent”). Time span was up to February 1st, 2024 by filters. The process of study selection involved screening titles and abstracts, followed by a comprehensive assessment of the full-text of studies that were potentially eligible.

### Eligibility Criteria

There were no limitations regarding the publication date, status, or language. We deemed papers or studies suitable for inclusion if they met the following criteria: (1) they involved human trials, including randomized controlled trials (RCTs) or observational trials; (2) they were reviews, case reports or series, editorials, letters, and comments; (3) they focused on PFO in children, child, pediatric, preschool, and adolescent populations; (4) they reported on the outcomes, diagnosis, management, and treatment of PFO. We excluded papers and studies that (1) involved animal experiments; (2) focused on PFO in adults; (3) were not full-text, such as conference abstracts.

## Results

### Study Selection and Characteristics

**Fig. 3:**
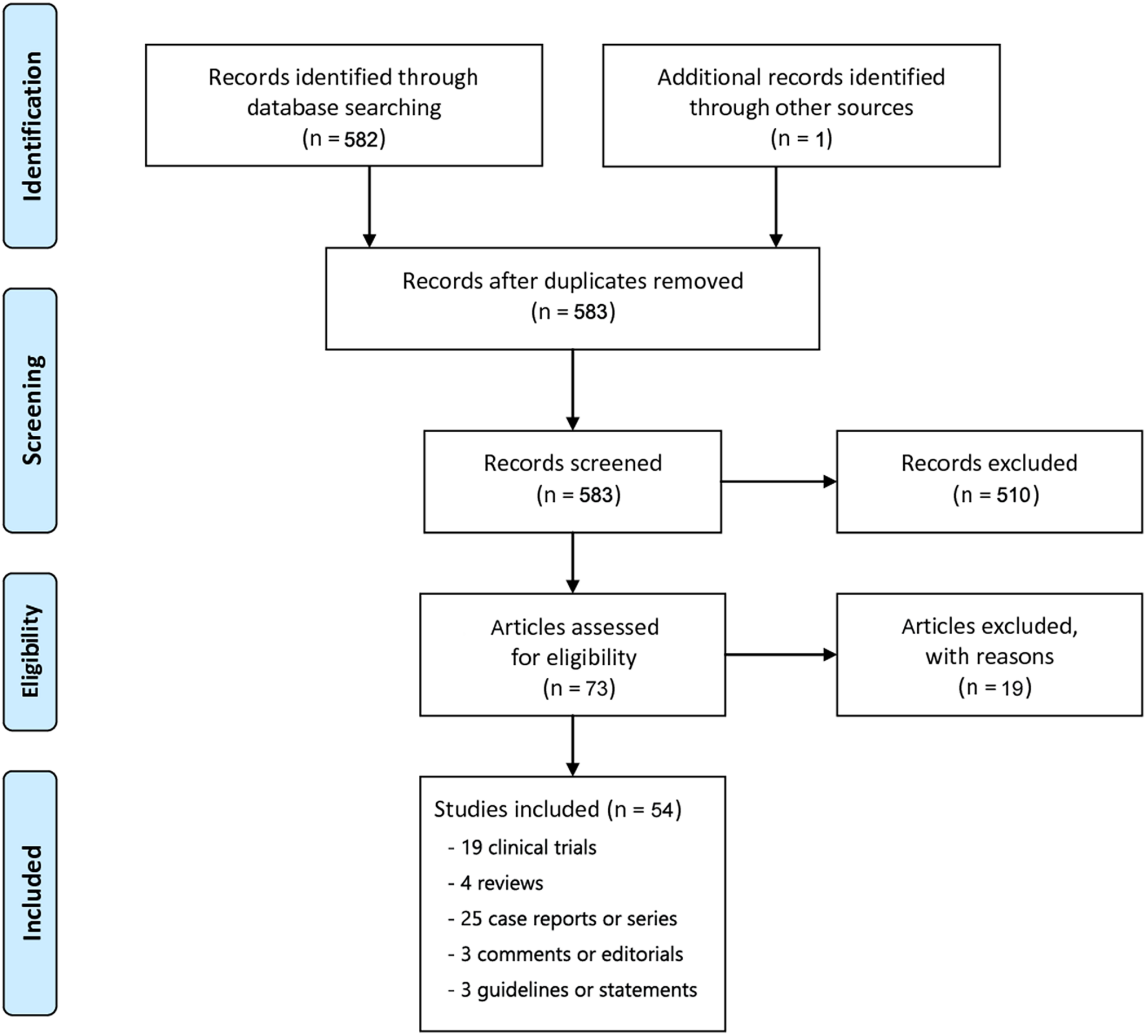
PRISMA flow diagram of study screening and selection

**Fig. 4:**
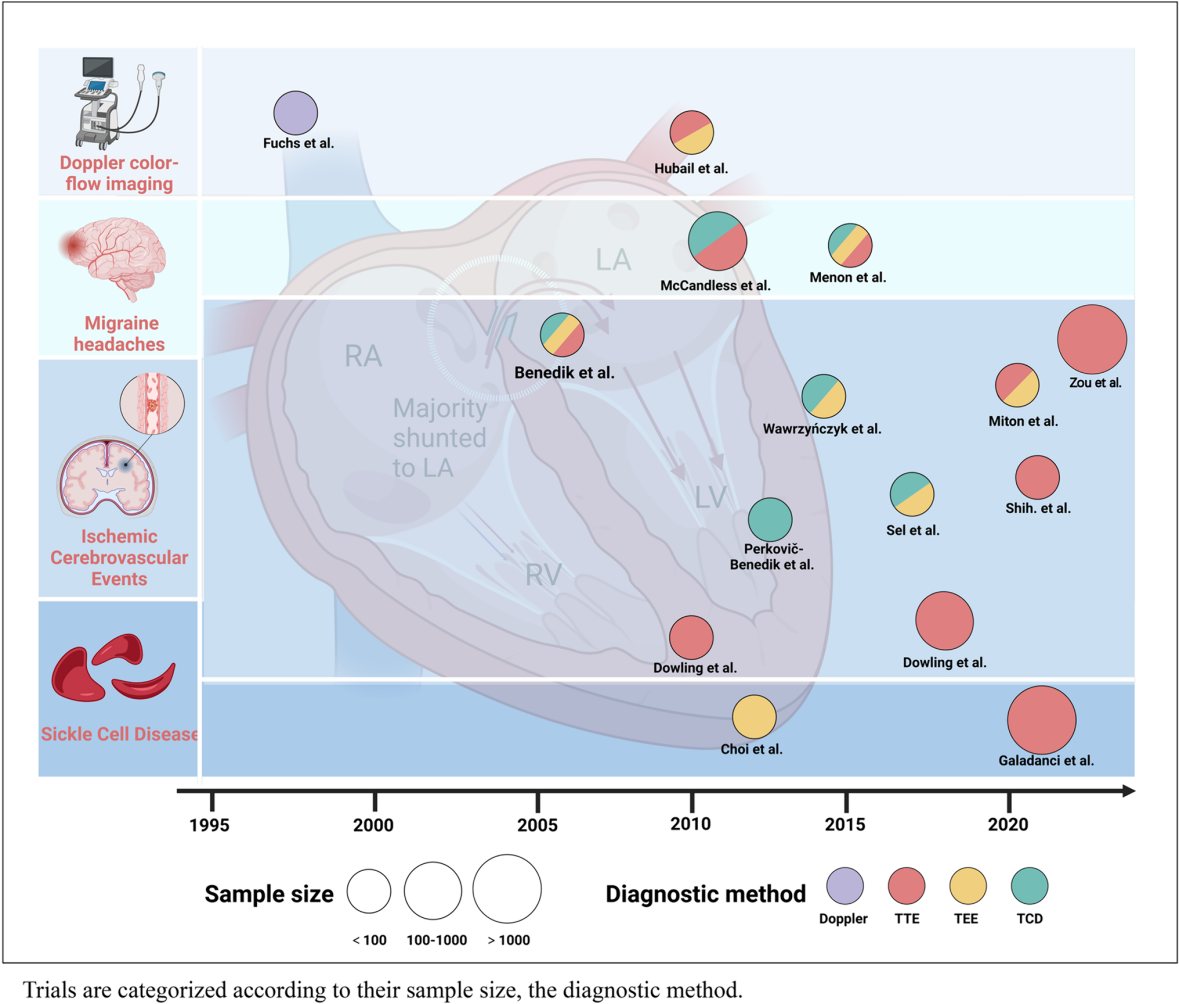
Previous investigations of patent foramen ovale (PFO) in pediatric patients

An extensive search was performed and a total of 583 articles were identified. Of which, 501 articles with the full-text were chosen for additional analysis. Ultimately, only 54 articles met the criteria for inclusion and exclusion, including 19 clinical trials, 4 reviews, 25 case reports or series, 3 comments or editorials, and 3 guidelines or statements (Fig. 3). The key features of the primary studies involving children with PFO are presented in Fig. 4 and Table 1.

**Table 1:** Previous main studies of patent foramen ovale (PFO) in children

	Population	Diagnosis of PFO or RLS	Results
Shih. et al. [8]	*N* = 25 in cryptogenic AIS *N* = 54 in known etiology AIS *N* = 209 healthy controls	TTE	PFO prevalence: *p* = 0.009 between the cryptogenic group and the known stroke etiology group; *p* = 0.03 between the cryptogenic group and control group
Benedik et al. [9]	N = 12 with ischemic stroke *N* = 6 with TIA	cTCD with VM TTE TEE	PFO plays an important role in CS in children; cTCD with VM plays an important role in PFO detection in children
Wawrzyńczyk et al. [10]	*N* = 2 with CS *N* = 5 with TIA	cTCD cTTE	Closure of PFO in children appears to be safe and effective; cTCD seems to be particularly suitable for children
Miton et al. [11]	*N* = 31 with stroke or TIA *N* = 10 with other reasons	TTE TEE	Closure of PFO in children appears to be safe and effective
Menon et al. [12]	*N* = 153 (migraine, other headache, visual symptoms, TIA, stroke-like symptom)	cTCD cTEE cTTE	Vast majority of children with PFO reporting significant improvement in their symptoms after closure; A strong placebo effect may exist
Sel et al. [13]	*N* = 10 with TIA *N* = 4 with stroke *N* = 3 with migraine	TCD TTE	PFO is a safe solution
Perkovič-Benedik et al. [14]	*N* = 23 with TIA *N* = 26 healthy controls	cTCD with VM	Paradoxical embolism (PE) may be important in pediatric patients with TIA
Choi et al. [16]	*N* = 32 with migraine *N* = 31 healthy controls	TTE	Migraine with aura is a clear predictor of PFO in children
McCandless et al. [17]	*N* = 109 with migraine	cTCD cTTE	PFO may contribute to the pathogenesis of migraine with aura in children
Zou et al. [18]	*N* = 1001 with syncope, palpitation, headache, dizziness, chest pain	TTE cTTE	PFO may not increase the risk of syncope in pediatric patients
Galadanci et al. [26]	*N* = 1414 with SCD	TTE	Overt stroke was more common in children with SCD combined PFO, but non-statistically; No evidence to support PFO was more common in children with sickle stroke
Dowling et al. [27]	*N* = 147 with SCA and stroke *N* = 123 without SCA or stroke	cTTE	The prevalence of potential RLS was significantly higher in the SCA + stroke group
Dowling et al. [28]	*N* = 40 with SCA and stroke	TTE	Intracardiac shunting could be a risk factor for stroke in children with SCD
Fuchs et al. [32]	*N* = 30 for neurosurgery in the sitting position	Doppler color-flow mapping	Doppler color-flow mapping could be a useful noninvasive technique to screen children scheduled for neurosurgery in the sitting position for the presence of a PFO
Hubail et al. [42]	*N* = 50	TEE with/without VM TTE with/without VM cTEE with/without VM cTTE with/without VM	cTTE is diagnostic of PFO in the majority of children

### PFO in Children with CS, TIA, and Migraine

The findings regarding PFO-associated stroke, TIA, and migraine in children were summarized. There was insufficient concrete evidence to substantiate the link between migraine with aura and PFO in children and adolescents [7], due to only a limited number of observational studies included in this study.

The results of a cohort study which assessed the prevalence of PFO in children with CS demonstrated a higher prevalence of PFO in the cryptogenic group in comparison to both the known stroke etiology group and the control group [8]. However, there was no significant disparity in stroke recurrence at the 2-year follow-up between children with and without PFO. Therefore, the author concluded that the optimal secondary preventive treatment for children with CS and PFO was still uncertain.

Benedik et al. conducted a retrospective study which consisted of 18 patients aged 2 to 17 years who suffered ischemic stroke or TIA to explore the involvement of PFO in CS among children between 2005 and 2007 [9]. Various diagnostic procedures were performed, including Transcranial Doppler (TCD) with agitated saline injection using the Valsalva maneuver (VM), transthoracic echocardiography (TTE), and transesophageal echocardiography (TEE). The significant role of PFO in CS among children was emphasized and the importance of contrast TCD (cTCD) with VM was highlighted for detecting PFO in children, as TEE examination required deep sedation, making VM impractical. This study suggested that percutaneous PFO closure should only be considered for children with CS and a high likelihood of PE.

A study which involved 7 patients aged between 10 and 15 who experienced CS or TIA was conducted in Poland from 2001 to 2011 to examine the lasting effects of cerebral events presumed to be caused by PE in pediatric patients [10]. This study indicated that CS resulting from PFO was infrequent in children and the closure of PFO in children was safe and effective which suggested that cTCD may be particularly well-suited for children.

A retrospective study involved 41 patients aged between 3 and 17.8 years who had experienced stroke, TIA, or other conditions like migraine, which was conducted in 9 pediatric centers in France between 2000 and 2019 to analyze the closure of PFO in children [11]. No delayed complications or instances of recurrent stroke were found after the closure of PFO conclude, which indicated that the closure of PFO in children seemed to be a secure and efficient procedure.

Menon et al. conducted the most comprehensive investigation to date on the outcomes of device closure of PFO in children, which was performed in 22 centers in Utah and Idaho from 1995 to 2010 [12]. A total of 153 patients, aged 7 to 19 years, who experienced migraines, non-migraine headaches, visual symptoms, TIA, and stroke-like symptoms, were enrolled. After undergoing PFO closure, complete resolution of their symptoms was reported and symptoms were reduced. The authors of the study suggested that this could be attributed to a reduction in the degree of shunting, as well as psychosocial factors or a placebo effect. A similar study was conducted by Sel et al. at a single center in Turkey from 2005 to 2014 [13].

Perkovič-Benedik et al. compared the prevalence and intensity of RLS in children with TIA with a control group using cTCD with VM [14]. The findings revealed a significantly higher occurrence of RLS in the TIA patients compared to the control, indicating the potential significance of PE in pediatric patients with TIA. Similar results were obtained when investigating children with arterial ischemic stroke (AIS) [15].

A study including 32 migraine patients with or without aura, and 31 normal controls was conducted in a single center in Korea from 2008 to 2011 to investigate the presence of PFO in children and adolescents with migraine [16]. The results revealed a significantly higher prevalence of PFO in individuals with migraine with aura compared to the other groups. The authors concluded migraine with aura can be considered a reliable indicator of PFO in children and adolescents. McCandless et al. conducted a study in Utah from 2008 to 2009, which explored similar aspects [17].

Zou et al. conducted a study to explore the relationship between PFO and syncope in pediatric patients. A total of 1001 pediatric patients aged 4–17 years, with symptoms such as unexplained syncope, palpitation, headache, dizziness, and chest pain, were assessed at a single center through TTE and contrast echocardiography to detect the presence of RLS. The findings of the multivariate logistic regression analysis revealed that PFO was not associated an increased risk of syncope [18].

Multiple cases of transcatheter closure of PFO in children with CS have been reported [19–23].

### PFO in Children with Sickle Cell Disease (SCD)

Sickle cell disease (SCD) is a hereditary monogenic hemoglobinopathy that is responsible for the majority of ischemic strokes in children. The risk of stroke in SCD patients is approximately 300 times higher than that of normal children. This increased risk can be attributed to various factors such as elevated levels of thrombin, abnormal fibrinolysis activation, fluctuating levels of anticoagulant proteins, and heightened platelet activation [24]. These factors contribute to the formation of blood clots, vaso-occlusion, and increased pressure in the right side of the heart. Consequently, young SCD patients with a PFO are more susceptible to developing RLS and PE, further increasing their susceptibility to stroke [25].

Galadanci et al. conducted a cohort study involved 1414 children with SCD to investigate the relationship between overt ischemic stroke and PFO in children with SCD. The results of the study revealed that children with SCD and PFO had a higher occurrence of overt stroke, although this association did not reach statistical significance [26].

Dowling et al. conducted a comparative investigation to showcase the higher occurrence of RLS in children with SCD and stroke. They performed contrast TTE (cTTE) on 147 children with SCD and stroke, as well as 123 children without SCD or stroke. The results revealed a significantly greater prevalence of potential RLS in the SCD plus stroke group compared to the control group [27]. The authors concluded that intracardiac shunting might pose as a risk factor for stroke in children with SCD as SCD patients are predisposed to thrombosis and elevations of right heart pressure, which could promote PE across the intracardiac shunt [28].

There have been reports of several cases where patients with both SCD and stroke had a PFO [29, 30]. Nevertheless, the optimal management strategy for these individuals with combined PFO remains unclear due to a lack of sufficient evidence.

### Other Studies or Cases of PFO in Children

Rodriguez et al. conducted a study to explore the potential mechanisms associated with cerebral microemboli during scoliosis surgery in adolescents. Their findings indicate that there is a risk of cerebral microembolization in young patients with PFO who undergo scoliosis surgery. Therefore, it is advisable to assess the feasibility of PFO closure as a preventive measure in these young patients [31].

Fuchs et al. conducted a study to examine the occurrence of PFO in children who were about to undergo neurosurgery while in the sitting position. PFO is known to be the primary cause of paradoxical air embolism during neurosurgical procedures in the posterior fossa in this specific position. The researchers discovered that Doppler color-flow mapping could be an effective noninvasive method to identify the presence of a PFO in children scheduled for neurosurgery in the sitting position [32].

Ossa-Galvis et al. reported the case of a remarkable teenage girl who excelled in swimming but experienced recurring chest pain, especially during strenuous activities. Seeking medical advice, she consulted a cardiologist who diagnosed her with a PFO. The cardiologist recommended that she avoid scuba diving due to the potential risk of stroke [33]. Similarly, Ali et al. reported a pediatric patient with a PFO and a history of migraine with aura who encountered a headache accompanied by neurological symptoms during a scuba diving lesson [34].

Platypnea-orthodeoxia syndrome is a rare medical condition characterized by positional dyspnea and decreased oxygen saturation while standing. Cetiner et al. reported a case of a 12-year-old girl diagnosed with platypnea-orthodeoxia syndrome associated with a PFO. After the PFO closure, the symptoms resolved effectively [35].

Myocardial Infarction (MI) is a rare occurrence in children. The main causes of MI in children include perinatal asphyxia in neonates and congenital anomalies of coronary arteries. There are also other potential causes such as Kawasaki disease, metabolic diseases, and coronary embolism [36]. Carano et al. documented a case of an 8-year-old girl who suffered from an acute MI. The angiographic examination of her coronary arteries revealed no abnormalities. However, further investigation uncovered a thrombophilic state caused by prothrombin G20210A mutation, as well as a PFO with RLS after a Valsalva maneuver, which was demonstrated by contrast TEE (cTEE). The authors proposed that a PE through the PFO led to the occurrence of MI [36].

Panagopoulos et al. reported an instance of cerebral hemorrhagic infarction in an 11-year-old individual. Subsequent investigations uncovered a thrombus in the right atrium, along with a PFO, pulmonary embolism, and deep venous thrombosis (DVT). The PE stemming from DVT through the PFO was identified as the underlying causal mechanism [37]. Filippi et al. presented a scenario of PE in a premature infant with central venous catheter-related DVT, where bilateral intraventricular clots were confirmed via neuroradiological assessment [38]. Loeffelbein et al. recorded a successful interventional closure of a PFO in a child supported by a biventricular assist device. The PFO was determined to be the source of arterial hypoxemia, and following the closure, the patient was able to be easily weaned off the respirator [39]. Henry et al. described a case of femoral artery embolus in a 12-year-old boy post-appendectomy, with a subsequent identification of a PFO [40].

### Diagnosing a PFO in Children

There are various techniques available for investigating a suspected PFO, such as TEE, cTEE, TTE, cTTE, TCD, and cTCD. In most cases involving children, cTTE has been proven to be effective in evaluating atrial septal integrity. TCD has the capability to identify gaseous microbubbles and small embolic material in the cerebral circulation. Typically, contrast is administered during TCD to generate intensity microembolic signals [41]. Although there is a lack of pediatric studies on TCD, its high sensitivity and specificity indicate that it could serve as a screening tool for a RLS in children who are able to perform the Valsalva maneuver. The Valsalva maneuver is commonly used for PFO assessment during TTE or TCD in adults. However, it may not be feasible for young children to properly execute this maneuver, although it can be utilized in older cooperative children and adolescents. TEE is considered the gold standard for investigating the embolic source in suspected stroke, as it offers detailed images of the posteriorly located left atrium and interatrial septum. Nevertheless, acoustic windows on TTE are generally superior in children compared to adults, and deep sedation may be necessary for TEE examination in children.

Hubail et al. conducted a research study to assess the precision of TTE or cTTE in detecting a PFO in children. They further compared the findings with TEE, which is widely regarded as the most accurate method [42]. The study included fifty pediatric patients aged over 1 year from a single center in Dallas between 2005 and 2006. The outcomes revealed that TTE exhibited a positive predictive value of 100% and a negative predictive value of 97%.

Latson proposed that cTCD is especially appropriate for the pediatric population because of its heightened sensitivity and the avoidance of sedation or anesthesia, which are frequently necessary for cTEE in children. It can serve as a screening tool for pediatric stroke patients. A negative cTCD outcome can reasonably rule out the necessity for a cTEE or cTTE, whereas a positive cTCD outcome still necessitates further verification to ascertain whether it is a PFO or another form of communication, such as a pulmonary arterio-venous malformation [43].

Kenny et al. considered that cTTE is the optimal choice for pediatric patients due to its ability to offer excellent transthoracic windows and enable children to execute sustained Valsalva during TTE. Additionally, they noted that with TCD, it might not be feasible to definitively determine if the PFO is the sole location of RLS [44].

Based on the evidence presented, cTTE emerges as the optimal examination for children due to its excellent transthoracic windows, the absence of sedation or anesthesia requirements, and ability to confirm the location of RLS.

### Management of PFO in Children

**Fig. 5:**
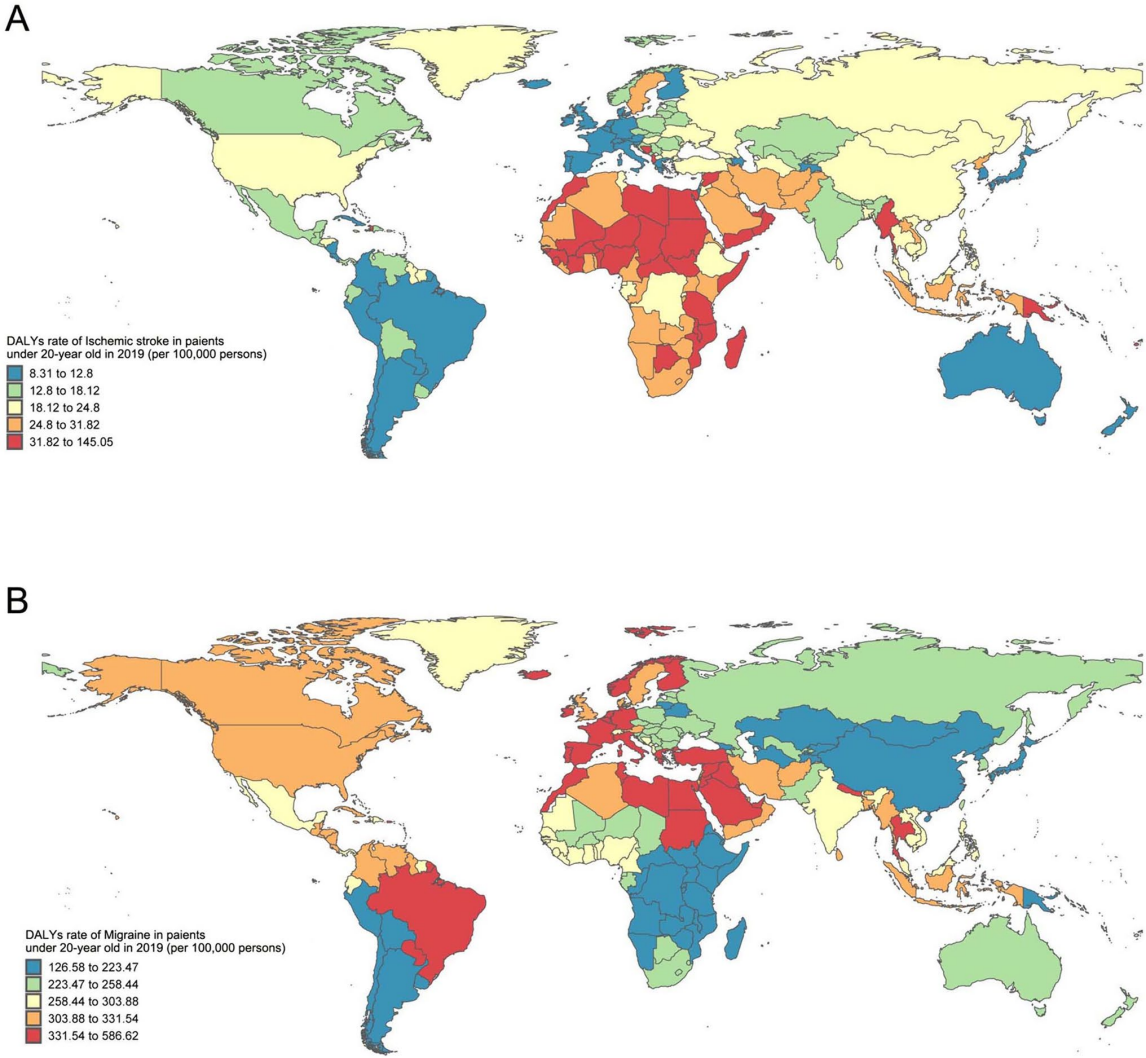
Disability adjusted life year rates of ischemic stroke (**A**) and migraine (**B**) in children

Despite the well-managed guidelines for PFO in adults [45, 46], there remains a dearth of pediatric data on PFO. The global burden of disease (GBD) has revealed that children also experience ischemic stroke and migraine (Fig. 5), which may be linked to other underlying conditions since children typically lack risk factors for primary stroke and migraine. The optimal approach to treating a PFO with RLS to prevent recurrent stroke in children is a topic of debate. PE through PFO is a diagnosis of exclusion, which complicates treatment decisions. The American College of Chest Physicians suggests PFO closure if an AIS is secondary to RTS in children [47]. On the other hand, the American Heart Association consensus statement does not provide a recommendation for PFO closure in preventing recurrent stroke in children due to insufficient evidence [48]. For healthy children with an incidental discovery of isolated PFO, no further treatment or follow-up is advised as there is currently no evidence of future complications associated with isolated PFO in children [46].

Currently, it is not recommended to perform PFO closure for primary stroke prevention in children, even though some lessons can be learned from adult trials in the fields of CS or TIA [49]. For migraine, PFO is more prevalent in children with migraine with aura [16, 17]. However, there is still a lack of evidence from RCTs to support medical treatment or PFO closure in children with migraines, though an observational study in children with migraine with aura did observe symptomatic improvement after PFO closure [12]. As for decompression sickness, there is currently no available pediatric data for young scuba divers, not do scuba diving could prevent stroke. However, if a patient wishes to continue diving and has a history of severe or recurrent decompression illness, a personalized interdisciplinary discussion is necessary. In the case of platypnea-orthodeoxia syndrome, device closure of PFO would likely benefit the patients. However, there is a lack of evidence specifically in the pediatric population.

To summarize, healthy children with an incidental finding of isolated PFO are advised against seeking further treatment or follow-up. In unique cases, a customized interdisciplinary conversation is essential. This is because there is currently no evidence suggesting future complications related to isolated PFO in children [46]. Merely extrapolating data from adults to pediatric patients is deemed inappropriate [50], underscoring the importance of conducting additional assessments via pediatric multicenter RCTs.

## Conclusion

This review suggested that the additional treatment was not needed as no evidence was provided for potential future complications linked to isolated PFO in children. For children with special circumstances related to PFO, a customized interdisciplinary discussion is essential prior to any medical intervention. Further multicenter research is required to establish evidence-based guidelines for managing PFO in pediatric clinical conditions.

## Abbreviations

AIS: Arterial ischemic stroke
cTCD: Contrast TCD
cTEE: Contract TEE
cTTE: Contrast TTE
CS: Cryptogenic stroke
DVT: Deep venous thrombosis
GBD: Global burden of disease
MI: Myocardial Infarction
PE: Paradoxical embolization
PFO: Patent foramen ovale
RCT: Randomized controlled trials
RLS: Right-to-left shunt
SCD: Sickle cell disease
TCD: Transcranial Doppler
TEE: Transesophageal echocardiography
TIA: Transient ischemic attack
TTE: Transthoracic echocardiography
VM: Valsalva maneuver
